# How mindfulness changed my sleep: focus groups with chronic insomnia patients

**DOI:** 10.1186/1472-6882-14-50

**Published:** 2014-02-10

**Authors:** Amber Hubbling, Maryanne Reilly-Spong, Mary Jo Kreitzer, Cynthia R Gross

**Affiliations:** 1 College of Pharmacy, University of Minnesota, Minneapolis, MN 55455, USA; 2 Center for Spirituality & Healing and School of Nursing, University of Minnesota, Minneapolis, MN 55455, USA; 3 College of Pharmacy & School of Nursing, University of Minnesota, Minneapolis, MN 55455, USA

**Keywords:** Chronic insomnia, Focus groups, Mindfulness, Content analysis, Self-efficacy, Social learning

## Abstract

**Background:**

Chronic insomnia is a major public health problem affecting approximately 10% of adults. Use of meditation and yoga to develop mindful awareness (‘mindfulness training’) may be an effective approach to treat chronic insomnia, with sleep outcomes comparable to nightly use of prescription sedatives, but more durable and with minimal or no side effects. The purpose of this study was to understand mindfulness training as experienced by patients with chronic insomnia, and suggest procedures that may be useful in optimizing sleep benefits.

**Methods:**

Adults (N = 18) who completed an 8-week mindfulness-based stress reduction (MBSR) program as part of a randomized, controlled clinical trial to evaluate MBSR as a treatment for chronic insomnia were invited to participate in post-trial focus groups. Two groups were held. Participants (n = 9) described how their sleep routine, thoughts and emotions were affected by MBSR and about utility (or not) of various mindfulness techniques. Groups were audio-recorded, transcribed and analyzed using content analysis.

**Results:**

Four themes were identified: the impact of mindfulness on sleep and motivation to adopt a healthy sleep lifestyle; benefits of mindfulness on aspects of life beyond sleep; challenges and successes in adopting mindfulness-based practices; and the importance of group sharing and support. Participants said they were not sleeping more, but sleeping better, waking more refreshed, feeling less distressed about insomnia, and better able to cope when it occurred. Some participants experienced the course as a call to action, and for them, practicing meditation and following sleep hygiene guidelines became priorities. Motivation to sustain behavioral changes was reinforced by feeling physically better and more emotionally stable, and seeing others in the MBSR class improve. The body scan was identified as an effective tool to enable falling asleep faster. Participants described needing to continue practicing mindfulness to maintain benefits.

**Conclusions:**

First-person accounts are consistent with published trial results of positive impacts of MBSR on sleep measured by sleep diary, actigraphy, and self-report sleep scales. Findings indicate that mindfulness training in a group format, combined with sleep hygiene education, is important for effective application of MBSR as a treatment for chronic insomnia.

## Background

An occasional sleepless night is a common complaint, but when difficulties initiating or maintaining sleep persist for months or years and cause daytime side effects such as fatigue and difficulty concentrating, the problem may be chronic insomnia [1]. About ten percent of US adults have chronic insomnia, with higher rates among women, the elderly, and people with physical or psychological illnesses [1,2]. Chronic insomnia is associated with poor health outcomes, increased health care costs and diminished quality of life, and although established therapies can improve sleep outcomes, most people do not obtain effective insomnia treatment [1,3]. Insomnia is estimated to reduce productivity in the US workforce by $63.2 billion [4], and another $32 billion is spent by US consumers in the ‘sleep market’ (e.g., sales of sleeping pills, sleeping masks, white noise machines, etc.) [5]. Clinical guidelines for treatment of chronic insomnia recommend multi-dimensional programs which combine relaxation training, stimulus control, sleep restriction and sleep hygiene (changing sleep habits) within a program of cognitive behavioral therapy (CBT-I) to address the dysfunctional beliefs and behaviors about sleep that perpetuate insomnia, however access to CBT-I is severely limited by a lack of sleep specialists [3,6]. Instead, most patients are untreated or take sedative hypnotics for years without addressing the underlying causes of their insomnia and despite troublesome side effects [7]. Amidst calls for innovative treatments and alternative delivery methods to expand access to care and accelerate improvements in treatment, mindfulness-based stress reduction (MBSR) has emerged as a promising therapy for chronic insomnia [8,9].

Patients with chronic insomnia are posited to be cognitively hyper-aroused and to experience mind-racing, worry or rumination when trying to fall asleep [8]. Mindfulness is maintaining present-centered, non-judgmental awareness with attitudes of acceptance and openness. The practice of mindfulness throughout the day is hypothesized to enable one to make intentional, skillful choices such as responding to stressors with appropriate actions, as opposed to acting on ‘automatic pilot’ with conditioned responses that can be emotionally arousing or harmful. At bedtime, mindfulness is hypothesized to disrupt rumination and worry, reduce verbal over-regulation and facilitate the dis-engagement necessary to fall asleep [10,11].

The MBSR program was developed by Kabat-Zinn to enable people to better adapt to the stressors of living with chronic illness [12]. Using MBSR to improve health outcomes is premised on the assumption that mindfulness will foster insight into psychological and behavioral sources of one’s suffering and thereby induce enhanced well-being or prompt action to engage in healthy behavior [13]. In Full Catastrophe Living, Kabat-Zinn suggests that “acceptance and mindfulness may help change dysfunctional beliefs about sleep, increase awareness of poor sleep hygiene habits, and discourage counter-productive behaviors of ‘trying too hard’ to sleep” [12]. He suggests mindful responses to sleep difficulties, including relaxation (using the body scan), exercise (yoga), and meditation as opposed to ‘catastrophizing’ about how a sleepless night will ruin the next day.

The classic MBSR program is taught by a certified teacher in 8-weekly, 2.5 hour group classes. The standard format also includes a day-long silent retreat and home practice requirements. Core components of the curriculum are: (1) experiential training in mindfulness meditation techniques such as focusing on the breath, sitting meditation and mindful Hatha yoga; (2) didactic information about stress and health; and (3) discussion about applications and adopting a personal mindfulness meditation practice.

MBSR has been shown to reduce symptoms of stress, anxiety and depression in clinical populations [14], enhance well-being in generally healthy adults [15] and to durably improve sleep outcomes in longitudinal studies of cancer patients [16], transplant recipients [17] and others [9]. Because sleep outcomes had shown large, significant treatment effect sizes in our MBSR trials to reduce symptom distress in transplant recipients [17,18], we conducted a randomized, controlled trial (RCT) to evaluate MBSR as a potential treatment for chronic insomnia. Thirty patients with primary chronic insomnia were randomized to either MBSR or nightly use of eszopiclone (Lunesta®), an FDA-approved sedative, in the Mindfulness versus Pharmacotherapy for chronic insomnia (MVP) trial [19]. MVP (ClinicalTrials.gov Identifier:NCT00515177) showed that MBSR could provide clinically meaningful reductions in insomnia symptoms as measured by sleep diaries, actigraphy and validated self-report sleep scales. Half of the MBSR patients met rigorous criteria for recovery from insomnia at the end of the study. Their sleep quality scores improved significantly, they were falling asleep 9 minutes sooner based on actigraphy, and they had high treatment satisfaction. None reported adverse effects. Whereas patients in the pharmacotherapy arm obtained similar benefits to sleep, treatment satisfaction scores were not high, and several reported adverse events. This trial provided initial evidence for the efficacy of MBSR as a viable treatment for chronic insomnia that has minimal risks, modest cost, and widespread availability.

To provide a rich context for interpretation of RCT results, focus groups were conducted with MBSR participants after their final trial follow-up. The purpose of the focus groups was to understand how mindfulness training influenced sleep and other aspects of patients’ lives. Focus groups were chosen instead of individual interviews because focus groups are a very efficient mechanism for generating rich data about a shared experience. The exchange among participants can provide immediate feedback about the salience and centrality of issues, as participants are able agree or disagree with each other and provide personal stories to illustrate their views.

Prior qualitative studies have explored the experiences of MBSR for patients with cancer [20-22], chronic low back pain [23], or substance abuse [24] and for healthy women [25,26], adolescents [27] and urban elderly [28]. To the best of our knowledge, this is the first qualitative study to address perceptions of MBSR’s effects from persons with chronic insomnia.

## Methods

### Participants

Patients diagnosed with primary chronic insomnia who completed MBSR in the MVP trial (N = 18) were eligible for the focus group study, and nine (8 women and 1 man, mean age 47, range 25–66 years, white race) participated. The MVP trial is described in detail by Gross et al. [19]. Briefly, patients meeting research criteria for chronic primary insomnia were randomized to MBSR or to 8 weeks of nightly use of a prescription sedative, and followed for 5 months. Chronic insomnia was defined as difficulty initiating or maintaining sleep despite adequate opportunity for sleep, with related daytime dysfunction on 3 or more nights a week for the past 6 months or longer [1,29], and based on a screening protocol which included a structured psychiatric interview, a one week screening sleep diary, and examination by a sleep physician. Those with insomnia considered due to a medical or psychiatric condition were excluded. The MVP trial and focus group study were approved by the University of Minnesota Institutional Review Board and Hennepin County Medical Center Review Board, and all participants provided written informed consent.

### Design

#### Conduct of the focus groups

Patients who attended at least 5 of the 8 MBSR classes (the benchmark for completion) were invited to attend a focus group approximately 5 months after their last MBSR class. Two focus groups (n = 5 and n = 4 participants) were held one year apart in 2008 and 2009. Groups were conducted according to the approach of Krueger and Casey [30]. The groups were convened by an experienced moderator in a quiet, private room and audiotaped. Each group lasted about two hours. The moderator followed a guide consisting of a short list of open-ended questions designed to elicit discussion relevant the study purpose (See Moderator's guide). Two assistants to the moderator took hand-written notes. At the end of each session, both assistants read summaries of their notes to the participants, and asked if all the main points were mentioned or if there were corrections or additions. Participants within each group had attended the same MBSR class.

#### Moderator’s guide

Moderator’s questions

1. Can you describe how your sleep routine was affected because of the class?

2. During the time you were attending the class, what did you do that was different than before?

3. Which practices are you still using and why?

4. How will you sustain the practice during the coming years? What can you do?

5. In what way did this class affect both your thoughts and your emotions?

6. If you had one minute to promote the class what would you say?

#### MBSR

MBSR was conducted using the standard format of 8 weekly, 2.5 hours classes, and a 6-hour silent retreat [31]. Meditation techniques included the body-scan, standing, sitting and walking meditations, and gentle Hatha yoga. Participants were given audio recordings of the guided meditations for home practice. Home practice expectations were 45 minutes of meditation per day at least 6 days a week for 8 weeks, followed by 20 minutes per day for 3 months. MBSR classes were led by a teacher who had completed basic and advanced MBSR instructor training [32]. The class-by-class outline in the 2003 MBSR instructor’s manual [33] was followed with the addition of assigning the chapter Sleep and Sleep Stress from Full Catastrophe Living [12] in week 2. To standardize knowledge of sleep hygiene in the MVP trial, a 10-minute presentation was given at the first MBSR class and booklets developed at the National Institutes of Health [34] distributed. Weekly phone calls from study staff monitored for adverse effects and encouraged meditation practice.

#### Analytic approach

Directed content analysis was used [35]. In this approach, an initial coding scheme is based on theory and relevant research findings. This initial coding scheme is iteratively revised and new codes or sub-codes created based on the data. This approach was chosen over a more purely inductive content analysis because prior work has examined the first-person experience of MBSR training, although not in the context of insomnia. The initial scheme was based on the open-ended questions about use of MBSR techniques and sleep posed by the focus group moderator (See Moderator’s guide).

Analysis began with reading the full transcripts to comprehend the group discussions as a whole, and then proceeded by comparing and categorizing codes across cases and across groups [30,35]. Two reviewers (A.H. and C.R.G.) first independently coded the transcripts, assigned codes to categories and combined categories into themes, then met to compare and discuss their coding and categorizations, reviewed the data again and held meetings with a third reader (M.R.-S.) to discuss inconsistencies and resolve differences. Transcripts were independently coded multiple times by each reviewer. This iterative process continued until a consensus set of themes that were inclusive of all participants was developed. Each reviewer completed a final categorization using the consensus themes, and results were combined into a final interpretation of the data.

## Results

Four major themes were identified from the focus group transcripts: the impact of mindfulness training on sleep and motivation to adopt a healthy sleep lifestyle; benefits of mindfulness on aspects of life beyond sleep; challenges and successes in adopting mindfulness-based practices; and the importance of group and teacher effects (Table 1). Each theme is described below, along with excerpts from the transcripts that exemplify the themes, and the source of each verbatim is indicated by first or second focus group (G1, G2).

**Table 1:** Themes and verbatims

**Theme**	**Sub themes**	**Selected verbatims**
Impacts on sleep	a. Impact of mindfulness on sleep	“I have been a lot more aware of everything I’m trying to do at night. And one of the things I try to do is I try not to eat or have as much beverage, and it did make a difference.” (G1)
	b. Motivation to adopt a healthy sleep lifestyle	
		“The body scan helped keep my mind from racing, so that I could just decompress and fall asleep” (G2)
		“[I am] making it a priority to do the things that we all know we are supposed to do, but we don’t necessarily do. Like not watching T.V. in bed, not eating chocolate at 7 o’clock at night.” (G2)
Benefits of mindfulness on aspects of life beyond sleep	a. Physical	“It’s like holistically positive” (G2)
	b. Emotional	“It’s low impact and I don’t get stiff like I used to sitting working long hours. I feel better. I [can] tell if I skip it - I’m going to hurt.” (G2)
	c. Relationships	
	d. Insights and changes in perception	
	e. Self-compassion	“I feel that when I started doing the body scan I was at a stable emotional level throughout the day.” (G1)
	f. Acceptance	
		“I’ve learned not to be so hard on myself. There’s a lot more things I could be doing than just being upset about myself.” (G1)
Challenges and successes in adopting mindfulness-based practices	a. Experiences with mindfulness-based practices	“enemy or best friend” (G1)
		“I loved when we got into the yoga. I never did the body scan again.” (G1)
	b. Incorporation of meditation practices into daily life	
		“there’s nowhere to go [and meditate] that someone isn’t going to be yelling ‘mom, mom’ ” (G1)
	c. Intentional choices and actions	
	d. Barriers to practice – time and environment	
		“Because I have difficulty falling asleep and difficulty staying asleep… I kept my little iPod thing or MP3 player by the bed.” (G2)
Importance of group support	a. Social support, validation and commitment to the group	“They’ve walked in our shoes” (G2)
		“I was like ‘wow, I’m not the only one.’ ” (G2)
	b. Learning from others	
	c. Relationship with MBSR teacher	“I don’t think I would have done it on my own… you really need the group to get you motivated.” (G1)
	d. Accountability to clinical trial staff	
		“It was just helpful to learn with other people.” (G2)

### Impact of mindfulness training on sleep and on motivation to adopt a healthy sleep lifestyle

Statements included in this theme described effects of mindfulness practices on falling or staying asleep and actions taken to make lifestyle changes which directly affected sleep.

a. *Impacts on sleep: “*just decompress and fall asleep” (G2)

Participants said that sleep initiation was easier, night-time awakenings were shorter, early awakenings were fewer and the quality of sleep was more satisfying. Several participants found practicing mindfulness techniques had an almost immediate impact on their ability to sleep. “Mine [my sleep] was almost immediately, positively impacted that I didn’t sleep longer, but I slept better. So, I woke up more refreshed even though I wasn’t sleeping more, and that happened for me very quickly.” (G2).

The body scan was singled out as a sleep-promoting technique that had great power to calm the mind and induce relaxation. One said “The body scan helped keep my mind from racing, so that I could just decompress and fall asleep,” (G2) and another said the body scan helps by “getting rid of all the other stuff that you might be thinking about… and I think that’s half the battle with relaxing and falling asleep.” (G1) A third participant said, “if I wake up in the middle of the night it [the body scan] seems to help me relax and get back to sleep. And a lot of times I’m back to sleep before I get past the left leg.” (G1) One participant described feeling “de-stressed” the moment she hears ‘and now’, the opening line of the body scan audiotape, and called her response “Pavlovian.” (G2) Long-acting benefits were also reported by participants who used the body scan with the intention of remaining awake in a state of meditative awareness. A participant who used the body scan in the morning said: “I feel as though instead of getting worked up about things throughout the day and then having it be difficult to come down to relax and sleep, I feel that when I started doing the body scan I was at a stable emotional level throughout the day. And I never get very emotional and then at the end of the day it’s much easier for me to calm down and relax.” (G1) Finally, participants found themselves better able to cope with insomnia when it did occur. “I think maybe the best thing I got out of it was that I’m not as stressed about not sleeping. I’m a lot more relaxed in cases that I don’t sleep.” (G1)

b. *Motivation to adopt a healthy sleep lifestyle:* “I had a major lifestyle change.” (G1)

Most participants experienced positive sleep impacts after making behavior changes that included both meditation practice and following sleep hygiene recommendations. Participants indicated that MBSR facilitated making behavioral changes, with comments about becoming more conscious and intentional about their sleep routine, and “making it a priority to do the things that we all know we are supposed to do, but we don’t necessarily do. Like not watching T.V. in bed, not eating chocolate at 7 o’clock at night.” (G2) Other actions included not accepting evening phone calls, and taking steps one participant referred to as “becoming very possessive of my winding down time.” (G1) Levels of motivation varied across participants, as reflected by choice of actions, intensity and persistence in meditating and engaging in sleep hygiene practices. One said, “I changed dramatically from the time I started the class. I changed everything. It wasn’t just the meditation part. I changed the time I went to bed, the time I got up. I tried to do all those things that were listed in that book [referring to Patlak [34]]. … So, I had a major lifestyle change… and I was sleeping great.” (G1) In reply to this comment, another participant expressed regret for not being more committed to making changes and reflected on how, for her, there was little sleep improvement.

Participants acknowledged that as meditation and sleep hygiene practices became more lax, benefits decreased or disappeared. One said, “I’ve actually noticed that if I don’t do the meditation I start getting a little more edgy.” (G1) Another spoke of a recent experience saying, “I couldn’t meditate during the vacation… And I noticed that the benefits left me….I came back home and here was the chatter all back again. ‘I shouldn’t have said that. Shouldn’t have done that. I said the wrong thing to that person.’…It was all back. And as I went to lay down and go to sleep I couldn’t go to sleep. And when I do the meditation that chatter goes away. And I can’t even say how it goes away, it just goes away. I lay down at night and I’m not chattery.” (G1) One participant motivated herself to keep up her meditation practice saying, “I just have to keep remembering and reminding myself how I feel when I do or don’t do it.” (G1).

### Benefits of mindfulness on aspects of life beyond sleep: “it’s like holistically positive” (G2)

Discussions revealed physical benefits, positive impacts on emotional regulation, relationships and communications with others, insight, acceptance, and self-compassion. The meditation technique most directly associated with physical benefit was yoga. Participants reported feeling physically better after yoga sessions, having increased flexibility, and the reduction of aches and pains, including low back pain. Several mentioned joining yoga classes, watching yoga on cable television or using yoga DVDs to maintain their practice after the end of the MBSR program. One participant said, “It’s low impact and I don’t get stiff like I used to sitting working long hours. I feel better. I [can] tell if I skip it - I’m going to hurt.” (G2).

Participants reported that mindfulness training had positive effects on their emotions and ability to deal with daily stressors. One described it as feeling “more peaceful and calm. It allows me to handle things that work me up better.” (G1) Another made the point that she felt more stable throughout the day but did not feel her positive emotions were “blunted.” (G1) Several participants described how their communications with family and co-workers were improved. One said “things just roll off me better [after doing the body scan regularly] and I don’t get upset as much. And I don’t yell at kids as much.” (G1) Another noted that mindfulness techniques helped her to let go of frustrating and stressful moments at work, and described how dealing with difficult co-workers became more manageable.

Participants described how MBSR classes opened their eyes to new insights. One participant admitted: “in the beginning, we talked about mindfulness. And it just struck me right away that at that point I wasn’t doing anything mindfully, nothing. I was multi-tasking and trying to keep everything in the air at once.” (G2) Another described learning to be more present-focused, saying “A lot of it was just reminding ourselves, slow down, focus. Don’t always be five steps ahead, ten steps ahead.” (G2) Another participant tied together the concepts of mindfulness and meditation practices to the importance of creating quiet moments: “I think there is value in being with yourself. Being in the moment. I think there is a lot of value to bringing yourself back to the here and now, and that’s what I get from walking meditation.” (G2).

For a few participants, the biggest lesson learned from MBSR was the ability to feel acceptance for their situation and demonstrate self-compassion. One busy mother said, “Where my life is right now, it’s [meditation and sleep hygiene practices] just almost impossible. I’ve got kids at home…there’s no way at 8 o’clock we can turn everything off. …there’s nowhere to go [and meditate] that someone isn’t going to be yelling ‘mom, mom’ or whatever. …And some of that, I think I’ve just learned to accept, that this is my life right now…and I do what I can.” (G1) Another acknowledged, “I’ve learned not to be so hard on myself. There’s a lot more things I could be doing than just being upset about myself.” (G1).

### Challenges and successes in adopting mindfulness-based practices: “enemy or best friend” (G1)

Participants spoke of finding particular meditation techniques useful and enjoyable. They also talked about techniques they did not like and why, and shared their experiences integrating mindfulness practices into their lifestyle. The body scan generated the most lively discussion. As summarized by one participant: “Body scan? That’s sort of everybody’s enemy or best friend at some point. Not everybody got to like it – to be their best friend – not everybody …. I remember I really hated it at first.” (G1) Common reasons for not liking to do the body scan were: “hard to stay still,” (G1) “too repetitive,” (G1) “too hard to keep my mind under control,” (G2) and “too long.” (G1) Many of the participants found yoga to be their preferred technique. One said, “I loved when we got into the yoga. I never did the body scan again. … I got so restless by the end [of the body scan]. When in the yoga I was doing something. I felt like I was accomplishing something.” (G1) There were also advocates for sitting meditation, walking meditation and mindful eating. Of sitting meditation, one participant commented: “It’s a good way to start the day. I’m in a rush to get out the door, and the bus comes and I sit there. I focus on just a short bus ride downtown.” (G2) To avoid conversation on the bus, she put on ear phones even when not listening to an audiotape. Other participants preferred sitting meditation before bedtime, as part of a pre-sleep winding down routine. Walking meditation was described as easy to incorporate into daily activities and a way to appreciate the outdoors.

Two kinds of barriers to practice - time commitment and the environment – were discussed. Finding time for daily practice was generally agreed to be challenging. One participant talked about “all or nothing” (G1) – feeling that it was not possible to practice unless one could commit the full 35 or 40 minutes for the body scan audiotape. Whereas some were able to practice in public (taking the bus, taking a walk), others felt they needed a quiet, private location where they would not be disturbed. Participants spoke of their practice being disrupted by going on vacation, having visitors and living with children.

### The importance of group sharing and support: “they’ve walked in our shoes” (G2)

Interactions among the members of the MBSR class had a powerful influence on participants’ willingness to try meditation techniques, adopt sleep hygiene practices and sustain a mindfulness practice. In the first focus group a participant noted, “My friends are like, ‘what do you mean you can’t sleep?’ They just don’t get *it*,” (G1) and in the second group one said, “I felt really validated in that first meeting where people were like ‘well, I’m here because I can’t sleep.’ I was like ‘wow, I’m not the only one.’ ” (G2) One participant described “energy” (G1) in the class with people bonding as they worked together on their insomnia. A participant from the first focus group said “the most beneficial thing for me was being in the group. You know, everyone doing it together. Hearing other people, where they’re at, was helpful.” (G1) In the second focus group, a participant said they learned something from everybody in the class, and another commented that “When I do stuff and it doesn’t work I find that thoroughly frustrating and that is hard … to have somebody else there saying ‘ it didn’t work for me either’ [is] *good*.” (G2) Another person said, “I don’t think I would have done it on my own… you really need the group to get you motivated.” (G1).

Participants perceived the MBSR teacher and study staff as committed, caring, genuine and empathetic. One participant explained “I think one of the things that made this class a success was really both [named MBSR teacher and study staff] being really committed and they understand where we were coming from. I mean they’ve walked in our shoes, and they know.” (G2) Another participant admitted that having her practice time monitored by study staff was motivating, saying “It helped knowing [the study staff] was paying attention…and I didn’t want to let her down. So, I’m still trying to do it.” (G1).

## Discussion

Two focus groups captured first-person accounts of insomnia patients’ experiences learning mindfulness techniques, applying their newly acquired meditation practices and knowledge to improve sleep, and describing personal benefits to sleep and other aspects of their lives. Participants told of falling asleep faster (sleep latency), returning to sleep sooner after night-time awakenings (reduced wake time after sleep onset) and awakening more refreshed. They did not report sleeping longer, but emphasized that being less stressed about their insomnia and better able to cope with occasional episodes of sleeplessness was a major benefit. They also mentioned benefits beyond sleep, including feeling more flexible and having fewer aches and pains, and being more calm and emotionally stable and as result, better able to relate to others and problem-solve at home and at work. The analysis revealed the process of gaining mindfulness skills to be quite challenging and the explanations for the sleep benefits obtained exposed the tightly woven interplay between motivation to make changes and the cognitive and physiologic impacts of those changes. These focus group findings complement and extend our previously published RCT findings of improvement on standardized scales of sleep quality, insomnia symptoms and dysfunctional sleep beliefs, and positive changes in objective and subjectively recorded sleep-wake parameters [19]. This evidence of mindfulness meditation’s benefit to chronic insomnia is important given insomnia’s negative health consequences [1,8] and huge monetary burden on society [4,5].

Overall, findings conformed to established models of health behavior change, and lend support to posited mechanisms of mindfulness for insomnia, including reduced arousal, rumination, worry and bed-time verbal over-regulation [10,11]. Findings were also congruent with the conceptual frameworks and hypothesized mechanisms responsible for the health benefits of mindfulness considered by Brown, Ryan and Creswell [13] and Shapiro et al. [36]: focused attention/awareness, exposure/acceptance, diminished emotional reactivity/increased emotional, cognitive and behavioral flexibility, insight/values clarification and enhanced self-regulation and self-management. There is as yet no consensus or proven mechanisms, but emerging evidence from experimental, clinical and imaging studies to support mechanisms of mindfulness have been recently reviewed by Hölzel et al. [37].

Participants were in the ‘action stage’ of readiness to make health behavior changes [38], as evidenced by enrolling in an insomnia treatment trial, and completing a 4-step screening process. Participants described how the MBSR program affected their sleep routine using words and examples congruent with established theories of health behavior change and the concepts of motivation, self-efficacy, outcome expectancies and social learning as articulated by Bandura [39]. Participants reported greater awareness of their own sleep-related behaviors, becoming more conscious and intentional about sleep behavior, and making some changes to their sleep routine a “priority.” These attitudes and actions are consistent with Brown and Ryan’s explanation of how mindfulness could build capacity to self-regulate behavior and motivate healthy coping in order to attain positive health outcomes [40]. Brown and Ryan noted that mindfulness enables recognition of automatic thoughts and behavior patterns, and is therefore a first step to disengaging from unhealthy habits. Similar to Fredrickson’s broaden and build theory [41], Brown and Ryan note that by being open, curious and attentive, mindful individuals can more effectively gather and interpret factual information to guide their health behaviors.

As participants started to experience improved sleep quality and positive impacts on their daily lives, they realized a strong connection between practice of mindfulness techniques and effects on emotional regulation. Consistent with the mechanisms proposed by Lundh and by Bootzin [10,11], participants regularly practicing mindful meditation techniques such as sitting meditation, the body scan or yoga reported being able to relax and to maintain feelings of calmness throughout the day, to avoid getting “worked up” or “stressed out” in responding to difficult people or problematic events, and to eliminate the “chatter” or mind-racing that previously impeded falling asleep at night. The inverse was also true: participants noted positive outcomes faded when mindfulness techniques were no longer practiced. These observations are consistent with experimental and physiologic evidence cited by Hölzel et al. [37] supporting emotional regulation as one of the main mechanisms responsible for the health benefits of mindfulness, and also congruent with Benson’s relaxation response [42].

Participants also gained new perspectives, letting go of the success or failure dichotomy of sleep or no sleep, perceiving and experiencing the value of being in the present with attitudes of awareness and acceptance. This led to enhanced self-efficacy, confidence and trust that one can cope with insomnia when it occurs. Experience provided a foundation for re-focusing outcome expectations beyond sleep to a more holistic view of benefits from stress reduction. This was especially important to busy parents, as they became to be more accepting of their situation and able to demonstrate self-compassion.

Our findings support and extend the sleep findings revealed by Morone et al. [23]’s content analysis of daily mindfulness practice logs written by 27 elderly patients during a clinical trial of MBSR for chronic low back pain. These investigators identified improved sleep as a major theme. Mindfulness may have had both direct effects on sleep and indirect effects through pain relief in these patients. Pain and poor sleep are frequently co-morbid; reduced pain and enhanced ability to cope with pain were also major themes in this study. Support for our findings of sleep benefit from “clearing the mind” can be found in verbatims reported in a recently published phenomenology study of the lived experience of MBSR training in 8 women with breast cancer conducted by Weitz et al. [22].

It is interesting to note that Morone et al. [23] termed sleep promotion a negative short-term side effect, because it interfered with maintaining awareness during meditation practice. Although participants in our study mentioned episodes of unintentionally falling asleep during guided meditations in class and during home practice, their reports were touched with irony and humor, and none ascribed a negative valence to this experience. Falling asleep during the body scan is a common occurrence, very familiar to MBSR teachers and described in Full Catastrophe Living [12].

Our findings strongly support adding sleep hygiene information to MBSR in order to optimize treatment impacts for patients with chronic insomnia. Because sleep hygiene education is the standard of care when prescribing sedative hypnotics, our trial protocol required both the MBSR and the pharmacotherapy treatment arms receive 10 minutes of in-person education and a sleep hygiene booklet from study staff (not the MBSR teacher) at the start of the trial. Focus group participants specifically mentioned taking actions to comply with the booklet’s recommendations for adopting a sleep-healthy bedtime routine and using the tips for responding mindfully to sleep problems described by Kabat-Zinn [12]. Having this information readily available was instrumental to those highly motivated to cure their insomnia. Although improved sleep outcomes after MBSR without added sleep hygiene has been reported [17,18], our focus group findings suggest adding sleep hygiene information may increase treatment effect sizes. It is noteworthy that others have begun to develop a program to integrate mindfulness training with CBT-I, the current gold standard non-pharmacological treatment for chronic insomnia, considering reduced arousal and rumination, and enhanced emotion regulation specific benefits of mindfulness training likely to make CBT-I more potent [43].

Shared experiences and support were also linked to the positive impacts of MBSR in our study. Similar findings have been noted in previous qualitative research about mindfulness training [20,21,44]. In Mackenzie et al. [21]’s analysis of interviews and a focus group with 9 cancer patients who were long-time attendees of a MBSR drop-in group, patients described the sense of validation and empowerment that arises from learning MBSR in a room full of people who were also cancer survivors, words echoed by our insomnia patients.

Our findings suggest that social learning is a key element responsible for the effectiveness of MBSR. Participants commented that being part of a group allowed for both observation and discussion. These interactions promoted connections between techniques and beneficial outcomes, and as a result increased motivation, self-efficacy and outcome expectations. Positive comments about the power of learning MBSR within a group were also elicited in focus groups with 8 breast cancer survivors by Dobkin [20]. Overall, these findings emphasize the importance of considering social learning and group effects when evaluating the mechanisms responsible for the treatment impact of the MBSR program.

Our findings about the challenges and successes of adopting a regular mindfulness meditation practice are congruent with the work of Kerr et al. [26] and Carroll et al. [24]. Kerr et al. conducted a longitudinal analysis of home practice diaries completed by 6 healthy women during their MBSR class. They found that each diary showed a trajectory of struggle to build a meditation practice, and levels of success varied. Carroll et al. identified the qualities of MBSR training useful to patients from a content analysis of stories written by patients who learned MBSR practices during a residential treatment program for substance abuse. These qualities were: the diversity of techniques/tools taught, the wide range of situations and settings where the tools can be employed, and the durability of the skills learned. These three factors, termed utility, portability and sustainability, were evident in the reports of our insomnia patients, who spoke of the flexibility of MBSR and told of using mindful approaches in communications and problem-solving at home and at work, and expressed gratitude for having acquired mindfulness skills for lifelong use.

### Strengths of the study

Focus groups generated first-hand accounts that provided a rich context for interpreting the results of a RCT of MBSR for chronic insomnia. This qualitative approach exposed factors which were not measured in the trial (e.g., use of sleep hygiene practices, amount of group support and social learning) as strongly linked to perceived sleep benefits. Modifications of the MBSR program that influence these factors are likely to impact the type and extent of sleep outcomes that can be achieved. This report provides information on the sleep impacts of MBSR in a group of patients who met rigorous diagnostic criteria for primary chronic insomnia. Although first-hand reports of the impact of mindfulness training on sleep have been reported in other populations, evidence of changes in this population strongly supports the clinical relevance of MBSR as a promising treatment for insomnia.

Several quality assurance steps were taken to ensure the legitimacy of results [30,45]. An experienced focus group moderator led both focus groups, using the same questions as prompts. When body language communications, such as nodding heads or shaking heads occurred, the moderator made a verbal comment (e.g., “I see nodding heads, can someone tell me more.”) to ensure that the audio transcript would reflect these sentiments. Credibility was demonstrated when participants agreed that the summaries read aloud by the moderator’s assistants were accurate reflections of the discussion. To promote reliability and reproducibility, transcripts were coded multiple times by two reviewers and inconsistencies resolved iteratively through discussion between these reviewers and a third party.

The characteristics of focus group participants were similar to all participants who completed MBSR in the MVP trial. There were no significant differences between those who did (n = 9) and did not participate (n = 9) in focus groups based on age (means = 46 and 45 years), race (100% white), gender (67% and 89% female) marital status (67% and 57% married), employed at least part-time (67% and 89%), duration of insomnia (means = 7 and 10 years), and number of medications at enrollment (means = 3.7 and 2.6). With respect to education, 44% of both focus group participants and non-participants had completed a graduate degree, and only one person had not attended college. Average baseline scores on self-report sleep scales [46-48] for focus group participants and non-participants were nearly identical (Pittsburgh Sleep Quality Index - 11.4 and 11.6; Insomnia Severity Index – 16.6 and 16.3; Dysfunctional Beliefs and Attitudes about Sleep – 5.4 and 5.7, respectively).

### Study limitations

Lack of gender and racial diversity are study limitations. Replication in a more diverse sample could yield additional findings. There is also the potential for selection bias; participants who had a poor experience with MBSR may have been less likely to attend a focus group. However, questionnaires completed by all subjects in the MBSR arm indicated high treatment satisfaction, so the risk of this type of bias is low. There is a risk that focus group participants over-reported benefits in an effort to please their MBSR teacher or to not disappoint the study investigators. To minimize this type of social desirability bias, the moderator and assistant moderators were not previously known to the participants. Directed content analysis may have set up expectations of what would emerge from discussion. This bias is acknowledged, as it stems directly from the purpose of the study (e.g., to learn how MBSR affected sleep), and was manifested through targeted questions posed by the moderator. These questions established boundaries for discussion and guided the initial coding scheme. However, the richness of the discussions which ensued revealed major themes which were not pre-determined, so we do not think this was problematic. However, it should be kept in mind that the subjects in this study completed a MBSR program within the context of a RCT for persons with chronic insomnia. Findings may not generalize to people taking MBSR on their own, to those taking MBSR without ancillary sleep hygiene information or to persons without insomnia. Finally, only two focus groups were held. Ideally, three or more focus groups should have been held to ensure data saturation. However, no new themes emerged in the analysis of the second focus group, which provides some confidence that data saturation was reached.

## Conclusions

Insomnia patients who completed MBSR were able to learn and use a variety of meditation techniques to fall asleep faster at bedtime, return to sleep sooner if awakened in the middle of the night, awaken more refreshed, and better cope with occasional episodes of sleeplessness. These findings validate and extend quantitative RCT results of improved sleep/wake parameters and recovery from insomnia following MBSR. Qualitative analysis found that during MBSR training patients are motivated to make healthy lifestyle behavior changes and are responsive to group effects. This MBSR program was delivered in group format, and sleep hygiene information was provided to augment the mindful sleep tips in Full Catastrophe Living [12]. Programs delivered in other formats and without sleep hygiene information may be less effective.

Mindfulness courses are available worldwide and are increasingly accessible. In the United States, there is a registry with over 600 trained MBSR teachers [32]. Classes are offered at wellness centers, in academic and corporate settings, and online. While MBSR courses are frequently considered educational and not a treatment, some insurance providers in the U.S. will pay some or all of the tuition, e.g. 50% reimbursement; in Canada many health plans reimburse the full cost. Workplace mindfulness programs have been delivered in major corporations like Apple, Google and General Mills [49]. Providing the traditional MBSR class on a sliding fee scale is becoming more common and costs vary by location, e.g. $250-400 Illinois, $350-550 Massachusetts, $500-700 Southern California, and some providers provide a limited number of scholarships.

Implications for future practice and policy include considering MBSR among the treatment options for primary chronic insomnia. MBSR influences the hyper-arousal and mind-racing that cause or perpetuate insomnia, and MBSR is not associated with side effects. In contrast, prescription sedatives do not address the underlying causes of insomnia, and have potential side effects that adversely impact quality of life and capacity to work in some patients. There are significant costs associated with treating insomnia, and MBSR is a promising approach that may be more accessible and cost-effective than other non-pharmacological options.

## Abbreviations

CBT-I: Cognitive behavioral therapy for insomnia; FDA: Food and drug administration; MBSR: Mindfulness-based stress reduction; MVP: Mindfulness versus pharmacotherapy for chronic insomnia trial; RCT: Randomized controlled trial; US: United States of America.

## Competing interests

The authors declare that they have no competing interests.

## Authors’ contributions

CRG conceived, designed and planned the study, conducted content analyses and co-wrote the manuscript. AH conducted content analyses and wrote the initial draft of the manuscript. MRS participated in the design, planning and conduct of the study including recruiting participants, preparing a moderator’s guide and summaries, adjudicated discrepancies among reviewers during the analyses and made revisions to the manuscript. MJK reviewed the manuscript. All authors read and approved the final manuscript.

## Authors’ information

MJK is the director of the Center for Spirituality and Healing at the University of Minnesota. The University of Minnesota is a major public university in the United States. The Center for Spirituality and Healing offers university classes and programs in a wide range of complementary therapies, including Mindfulness-based stress reduction.

## Pre-publication history

The pre-publication history for this paper can be accessed here:

http://www.biomedcentral.com/1472-6882/14/50/prepub
